# Asymmetrical degenerative marrow (Modic) changes in cervical spine: prevalence, correlative factors, and surgical outcomes

**DOI:** 10.1186/s13018-018-0807-0

**Published:** 2018-04-16

**Authors:** Xianda Gao, Jia Li, Yiqing Shi, Shaoqing Li, Yong Shen

**Affiliations:** Department of Orthopedic Surgery, The Third Hospital of Hebei Medical University, No. 139 Ziqiang Road, Shijiazhuang, 050051 China

**Keywords:** Cervical spine, Modic changes, Prevalence, Correlative factors, Surgical outcomes

## Abstract

**Background:**

The current study aimed to discuss the prevalence and surgical outcomes of the asymmetrical Modic changes and identify its correlative factors by multivariate logistic regression analysis.

**Methods:**

Two hundred seventy-eight patients with single-level Modic changes and nerve compression symptoms were reviewed retrospectively from January 2008 to January 2015. 1.5-T MRI was performed to determine the Modic changes. Multivariate logistic regression analysis was used to identify the correlative factors of asymmetrical Modic changes. Surgeries were performed according to the surgical indications. The outcomes were recorded by Japanese Orthopaedic Association (JOA) score, Neck Disability Index (NDI) score, and recovery rate.

**Results:**

Asymmetrical Modic changes were observed in 76 patients (27.34%) with 4 type 1, 69 type 2, and 3 type 3. C5/6 was the most frequently affected segment with 39 patients showing signal changes on MRI. Statistically significant difference was showed in conservative rehabilitation rate between two groups (*p* = 0.043). Multiple logistic regression analysis identified disc herniation and neurological symptoms as correlative factors of asymmetrical Modic changes, and the adjusted odds ratios (95% CI) were 2.079 (1.348–3.208) and 0.231 (0.143–0.373) respectively. No statistically significant difference was found in JOA scores and NDI scores between the two kinds of Modic changes.

**Conclusions:**

C5/6 was the most commonly affected level by Modic changes. Disc herniation and nerve root compression symptom were more closely correlated with asymmetrical Modic changes than conventional Modic changes. Asymmetrical Modic changes indicated poor result in conservative treatment; however, the final operation rate was similar between the two kinds of Modic changes. The outcomes of surgical treatment were satisfactory both in patients with asymmetrical Modic changes and conventional Modic changes.

## Background

**Fig. 1:**
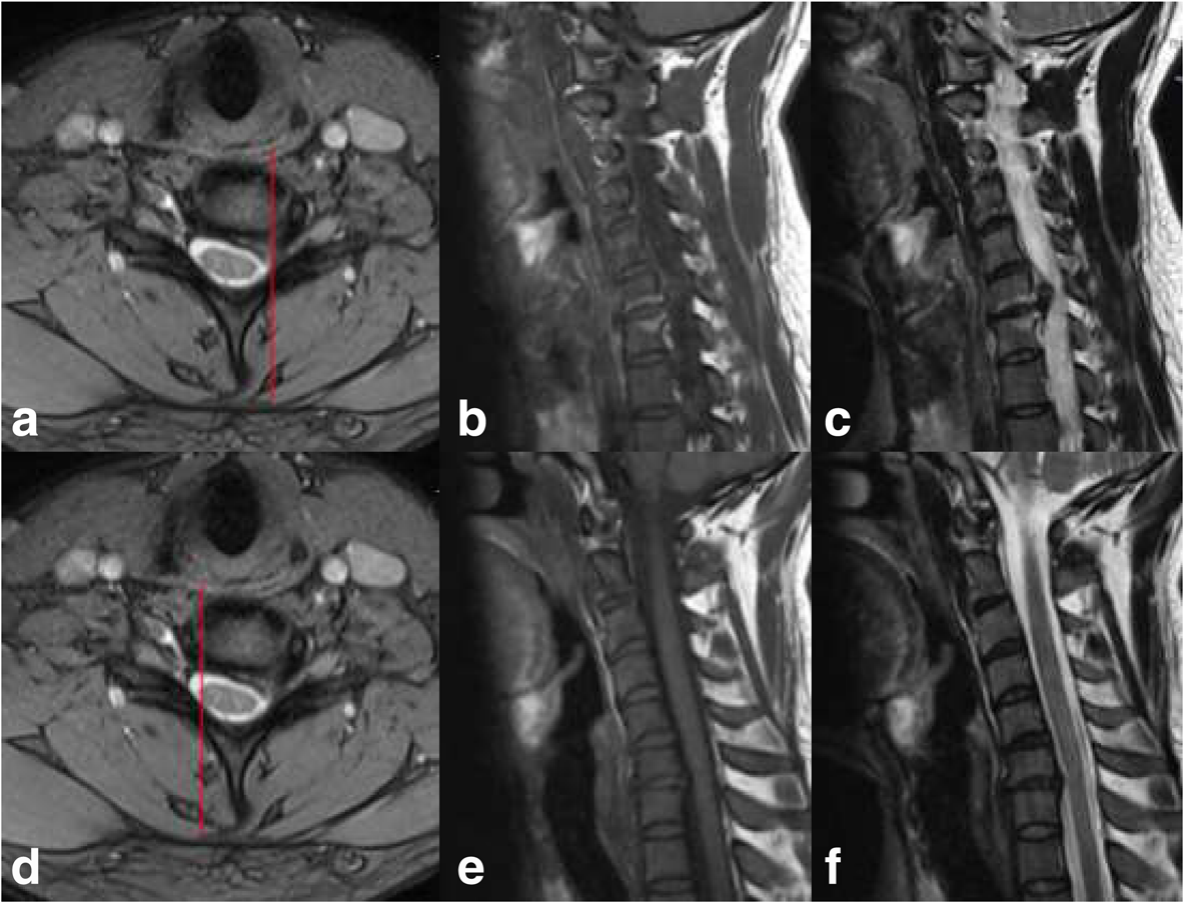
Asymmetrical Modic changes. **a** The axial view of MRI and the red line was the location of **b** and **c**. **b**, **c** Both T1 and T2 hyperintense on the left side of C6-7. The red line in **d** was the location of **e** and **f**. **e**, **b** No signal changes were found on the right side of C6-7, which indicated MCs in C6-7 were asymmetrical and unilateral

**Fig. 2:**
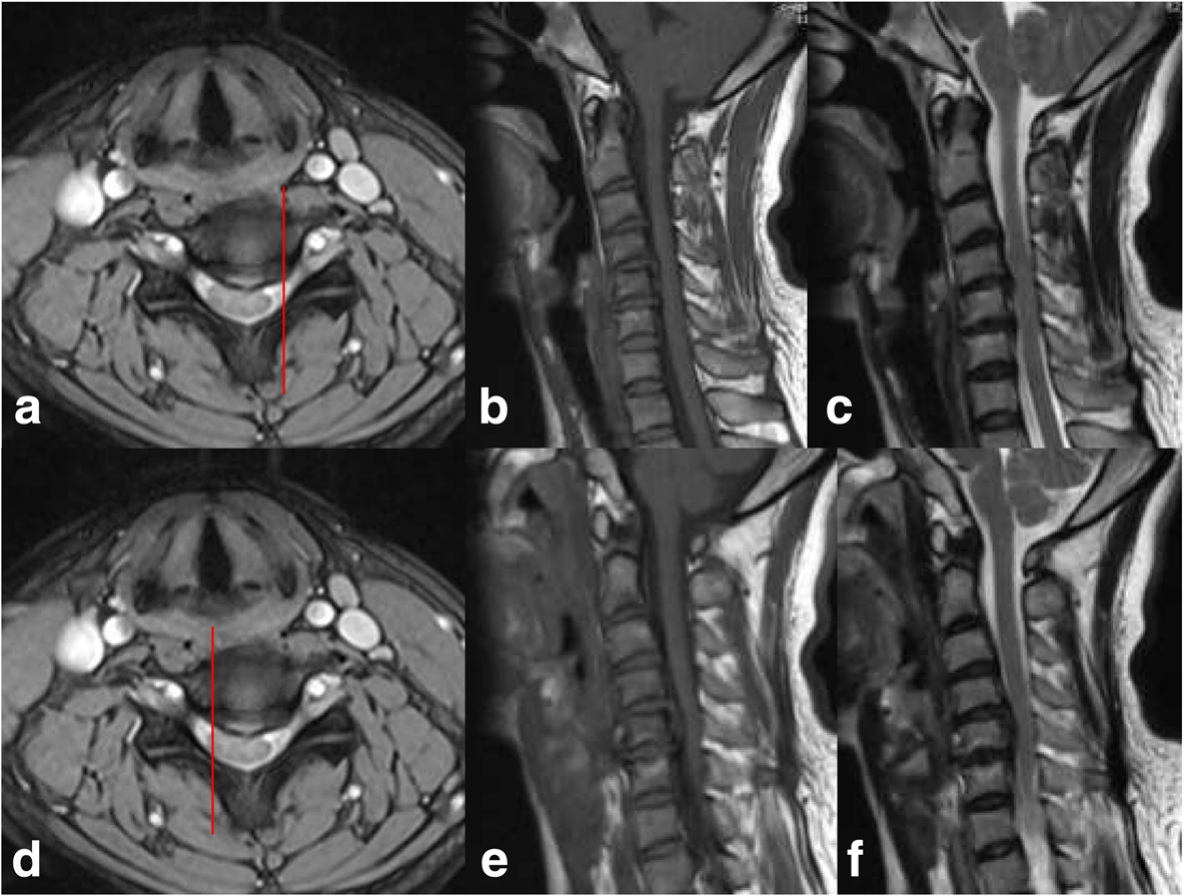
Conventional Modic changes. **a**, **d** The axial view of MRI and the red lines were the location of **b** and **c** in **a** and **e** and **f** in **d**. Whether on the left side of on the right side, T1 and T2 hyperintense always existed, which indicated the signal changes throughout the whole endplant

Modic changes (MCs) are signal changes of vertebral endplant and subchondral bone marrow on magnetic resonance imaging (MRI), which were first reported by de Roos [1] on lumbar spine. Modic MT [2, 3] classified the signal changes into three types according to signal intensity on T1-weighted and T2-weighted imaging: type 1 was T1 hypointense and T2 hyperintense; type 2 was both T1 and T2 hyperintense; and type 3 was hypointense in both T1 and T2. MCs in the lumbar spine were discussed in lots of studies; however, MCs in the cervical spine were proposed after a period of time. MCs in cervical spine were first epidemiologically reported by Peterson CK in 2007 [4] that MCs were found in 19 of the 118 patients (16%) with the dominant type 1. However, the subsequent studies showed type 2 was the most frequently observed [5–8]. Asymmetrical MCs were unilateral degenerative marrow changes adjacent to the intervertebral foramen (Fig. 1), which were first proposed in the current study. The concept of asymmetrical MCs was distinguished from the conventional MCs which were throughout the whole endplant (Fig. 2). The purpose of this study was to discuss the prevalence of the asymmetrical MCs and identify its correlative factors by multivariate logistic regression analysis. In addition, anterior or posterior cervical surgeries were performed in a part of the patients according to the surgical indications with minimum 2-year follow-up, and the outcomes of the patients were compared between two kinds of MCs.

## Methods

### Patients

Two hundred seventy-eight patients, including 157 females (56.47%) and 121 males (43.53%), with single-level MCs and nerve compression symptoms were reviewed retrospectively in the current study from January 2008 to January 2015. Exclusion criteria comprised trauma, infectious diseases, tumors involving cervical spine, inflammatory changes, congenital malformation, and scoliosis and patients who had undergone radiotherapy or cervical surgery. Surgical intervention was performed with the following indication: (1) severe pain of cervical spondylotic radiculopathy which affected sleep, (2) moderate or severe cervical spondylotic myelopathy, (3) with neurological deficit, muscular atrophy, and (4) conservative treatment for 3 months with poor effect. Conservative treatment included rest, medication, physiotherapy, collar, and traction. Anterior cervical diskectomy and fusion (ACDF) were performed in patients with compression less than three levels and without ossification of posterior longitudinal ligament (OPLL). Laminectomy with lateral mass screw fixation was performed in patients with compression greater than three levels and with OPLL or compression mainly resulting from ligamentum flavum. All the surgeries were performed by the same senior surgeon. The mean age at the operation was 52.27 ± 7.59 years, and the follow-up period after surgery was 2.56 ± 0.79 years. Patients’ age, gender, Modic type, disc herniation, disc degeneration, neurological symptoms, axial symptom, smoking or not, surgery rate, and surgical methods were collected as potential correlative factors of asymmetrical MCs. Neurological symptoms included nerve root compression symptom and spinal cord compression symptom. Axial symptom was defined as neck or/and shoulder pain and stiffness. It was blinded between the assessors who reviewed the MRI scans and the assessors who collected the clinical data of patients.

### Imaging and clinical evaluation

MCs were determined by cervical 1.5-T MRI (Siemens MAGNETOM Symphony), and three-type classic classification was applied, which meant mixed types were not considered. Disc herniation at the level with MCs was assessed on T2-weighted sagittal MRI according to modified Matsumoto’s classification [9, 10] (Table 1). Disc degeneration at the level with MCs was divided into five grades by four indicators according to Miyazaki classification [11] (Table 1).

**Table 1:** Miyazaki classification and Matsumoto’s classification

Miyazaki classification				
Grade	Nucleus signal intensity	Nucleus structure	Distinction of nucleus and annulus	Disc height
I	Hyperintense	Homogenous, white	Clear	Normal
II	Hyperintense	Inhomogenous with horizontal band, white	Clear	Normal
III	Intermediate	Inhomogenous, gray to black	Unclear	Normal to decreased
IV	Hypointense	Inhomogenous, gray to black	Lost	Normal to decreased
V	Hypointense	Inhomogenous, gray to black	Lost	Collapsed
Matsumoto’s classification				
Grade				
I	Disc bulging beyond the posterior margin of the vertebral body			
II	Disc bulging over half of epidural space			
III	Disc protrusion bordering the spinal cord			
IV	Disc protrusion compressing the spinal cord			

Japanese Orthopaedic Association (JOA) score was used to assess the functional neurological status before surgery and at the final follow-up visit. The recovery rate was calculated by JOA score as follows: (postoperative JOA score − preoperative JOA score)/(17 − preoperative JOA score) × 100%. The axial symptom was assessed by Neck Disability Index (NDI) before surgery and at the final follow-up visit. The patients were followed up for minimum 2 years.

### Statistical analysis

SPSS program (version 22.0; SPSS Inc., Chicago, IL, USA) was used for the statistical evaluation. Quantitative date was tested by Student’s *t* test or Mann–Whitney *U* test, as appropriate. Qualitative date was tested by chi-square test. The correlative factors were determined by multivariate logistic regression analysis with adjusted odds ratios (ORs) and 95% confidence intervals (CIs). The factors with *p* < 0.05 in univariate analysis were selected into the multivariate logistic mode. Dummy variable was used for multi-categorical data in multivariate logistic regression analysis. *p* value < 0.05 was considered statistically significant difference.

## Results

### Prevalence

Twenty-one type 1 (7.55%), 244 type 2 (87.77%), and 13 type 3 (4.68%) MCs were observed in the total 278 patients. C5/6 was the most frequently affected segment by MCs with 122 patients (43.88%) showing signal changes on vertebral endplant and subchondral bone marrow (Table 2). The second and third most frequently affected segments by MCs were C6/7 (65 patients 23.38%) and C4/5 (48 patients 17.27%) respectively (Table 2). There were no MCs observed in C2/3 level. Asymmetrical MCs were observed in 76 patients (27.34%) with 4 type 1 (5.26%), 69 type 2 (90.79%), and 3 type 3 (3.94%). C5/6 was also the most frequently affected segment by asymmetrical MCs with 39 patients (51.32%) showing signal changes on MRI (Table 2). No significant differences were found in Modic types (*p* = 0.621) and segments (*p* = 0.461) between asymmetrical MCs group (group AM) and conventional MCs group (group CM).

**Table 2:** Prevalence of Modic changes in each segment

Segment	Asymmetrical MCs		Conventional MCs		Total
	Segment number	Proportion	Segment number	Proportion	
C2/3	0	0	0	0	0
C3/4	9	11.84%	34	16.83%	43 (15.47%)
C4/5	12	15.79%	36	17.82%	48 (17.27%)
C5/6	39	51.32%	83	41.09%	122 (43.88%)
C6/7	16	21.05%	49	24.26%	65 (23.38%)
Total	76	100.00%	202	100.00%	278 (100.00%)

### Correlative factors

Statistically significant difference was not found in age, gender, disc degeneration, axial symptom, smoking, surgery rate, and surgical method between group AM and group CM (Table 3). The average modified Matsumoto’s grade of disc herniation in group AM was 3.18 ± 0.67, which was greater than that (2.83 ± 0.69) in group CM (*p* < 0.001) (Table 3). But the grade of disc degeneration was similar between the two groups (*p* = 0.209). In group AM, there were 55 patients suffering nerve root compression symptom, 10 patients suffering spinal cord compression symptom, and 11 patients suffering mixed symptom, while these number of patients in group CM were 112, 64, and 26 respectively (*p* = 0.007) (Table 3). Fifty-one patients in group AM chose the conservative treatment, and finally, surgical intervention was avoided in 24 patients (47.06%) with remission or disappearance of symptoms. Initially, 116 patients chose the conservative treatment, and 74 patients (63.79%) felt symptomatic improvement and avoided surgery. A total of 52 patients (68.42%) in group AM and 128 patients (63.37%) in group CM underwent surgical treatment. Statistically significant difference was showed in conservative rehabilitation rate between the two groups (*p* = 0.043), while the final surgery rate was similar (*p* = 0.432) (Table 3).

**Table 3:** Comparison of patient characteristics between group AM and group CM

Variable	Group AM (*n* = 76)	Group CM (*n* = 202)	*p* value
Age (years)	51.66 ± 6.96	52.50 ± 7.82	0.414^1^
Gender			0.287^2^
Male	37	84	
Female	39	118	
Modic type			0.621^2^
Type 1	4	17	
Type 2	69	175	
Type 3	3	10	
Disc herniation	3.18 ± 0.67	2.83 ± 0.69	< 0.001^3^
Disc degeneration	3.45 ± 0.74	3.29 ± 0.91	0.209^3^
Neurological symptoms			0.007^2^
Nerve root compression	55	112	
Spinal cord compression	10	64	
Mixed symptoms	11	26	
Axial symptom			0.124^2^
Yes	22	78	
No	54	124	
Smoking			0.371^2^
Yes	28	63	
No	48	139	
Surgery rate	68.42%	63.37%	0.432^2^
Conservative rehabilitation rate	47.06%	63.79%	0.043^2^
Surgical method			0.226^2^
Anterior approach	85	168	
Posterior approach	11	34	

^1^Independent *t* test

^2^Chi-square test

^3^Mann–Whitney *U* test

Univariate logistic regression analysis showed greater modified Matsumoto’s grade of disc herniation (*p* < 0.001) and nerve root compression symptom were related to the asymmetrical MCs. Multiple logistic regression analysis showed the adjusted OR (95% CI) of disc herniation and neurological symptoms were 2.079 (1.348–3.208) and 0.231 (0.143–0.373) respectively (Table 4). The correlation was closer between asymmetrical MCs and nerve root compression symptom than spinal cord compression symptom (*p* < 0.001) or mixed symptoms (*p* < 0.001).

**Table 4:** Correlative factors for asymmetrical Modic changes: multiple logistic regression analysis

Variable	Adjusted odds radio	95% confidence interval	*p* value
Disc herniation	2.079	1.348–3.208	0.001
Neurological symptoms	0.231	0.143–0.373	< 0.001

### Surgical outcomes

JOA scores and NDI scores were recorded in 52 patients in group AM and 128 patients in group CM who underwent surgical treatment. The average follow-up period was 2.56 ± 0.78 years. Preoperative JOA scores and ODI scores in group AM were 7.92 ± 2.21 and 21.67 ± 7.76 and in group CM were 7.49 ± 2.31 and 20.83 ± 8.90, respectively. JOA scores (12.66 ± 1.97) were significantly increased (*p* < 0.001) after surgery and ODI scores (8.13 ± 5.91) was significantly decreased (*p* < 0.001) after surgery in Group AM. The similar results showed in group CM. The recovery rate in group AM and group CM was 54.61% ± 12.01% and 53.62% ± 13.33%, respectively. No statistically significant difference was found in preoperative JOA scores (*p* = 0.180), preoperative ODI scores (*p* = 0.269), final follow-up JOA scores (*p* = 0.588), and final follow-up NDI (*p* = 0.165) scores between the two groups (Table 5).

**Table 5:** Clinical evaluation: JOA scores and NDI scores

	JOA			NDI		
	Preoperative	Final follow-up	*p* value	Preoperative	Final follow-up	*p* value
Group AM	7.92 ± 2.21	12.66 ± 1.97	< 0.001	21.67 ± 7.76	8.13 ± 5.91	< 0.001
Group CM	7.49 ± 2.31	12.42 ± 1.88	< 0.001	20.83 ± 8.90	9.06 ± 6.56	< 0.001
*p*	0.180	0.269		0.588	0.165	

## Discussion

There were many studies investigating the MCs in lumbar spine, but only a few studies discussing the MCs in cervical spine. The classification of MCs relied on the signal intensity on T1 and T2 MRI. MCs were considered to be a kind of inflammatory change in vertebral endplant and subchondral bone marrow. Inflammatory mediators such as interleukin, prostaglandin E2, PGP 9.5, and TNF were proved to be relevant to MCs, especially type 1 [12–15]. MCs type 1 was the active stage of inflammation with more inflammatory mediators than type 2 or type 3. Histological changes in type 2 was that the yellow fatty marrow took the place of vertebral endplates and in type 3 was sclerotic bone took the place of vertebral endplates. With the progression of disease, MCs type 1 would convert to types 2 or 3 which were the stable stage of inflammation. The etiology of MCs was still controversial. Some studies revealed inflammatory change was caused by low grade anaerobe, especially propionibacterium acnes, and the subsequent antibiotic therapy was effective [16–18]. However, Wedderkopp et al. [19] took biopsies of affected vertebra from 24 patients with MCs type 1 by a strict aseptic procedure and incubated bacteria. The results showed none of the samples cultured anaerobe, only two samples cultured aerobe, and the subsequent antibiotic therapy was ineffective. Another studies also indicated there was no association between MCs and low-grade infection [20]. The pathophysiology of MCs was complex, and further studies would be performed to confirm whether the MCs were bacterial inflammation.

Signal changes of vertebral endplant and subchondral bone marrow in cervical spine was first reported by Peterson et al. [4] in 19 of the 118 patients (16%). The subsequent studies showed the incidence rate of MCs in cervical spine was ranged from 3 to 40% resulted from different inclusion criteria [2, 3, 6, 7, 10, 21–23]. The highest incidence rate was 40.4% which was reported by Mann et al. [5] in 426 patients whose ages were more than 50 years, while the lowest incidence rate was reported by Matsumoto et al. [22] as 3% in 4 of the 133 patients who suffered whiplash injuries in the initial. MCs type 2 was dominant type in most studies [2, 3, 6, 7, 10, 21–23] except for Peterson [4] who reported type 1 was the most frequently found. MRI was the only diagnostic method for MCs and its typing diagnosis. However, it was reported [24] that magnetic field intensity had an influence on diagnosis of MCs and its typing. Bendix et al. [24] found that high-filed (1.5T) MRI diagnosed more MCs than low-filed (0.3T) MRI. He also found that high-filed MRI diagnosed twice as many MCs type 2 as low-filed MRI, while low-filed MRI diagnosed three to four times Modic type 1 changes compared with high-filed MRI. In the current study, with 1.5T MRI, MCs type 1 accounted for 7.55%, type 2 accounted for 87.77%, and type 3 accounted for 4.68%.

Asymmetrical MCs occurred on one side of endplant and subchondral bone marrow, often accompanied by disc herniation and nerve root compression symptom. Matsumoto et al. [21] found MCs in cervical spine were significantly associated with numbness or pain in the arm in his 10-year follow-up study. Our study only enrolled patients with single-level MCs to avoid the mixed symptoms resulted from mixed kinds of MCs and mixed types which appeared on one patient. Compared with conventional MCs, the degree of disc degeneration is similar in asymmetrical MCs; however, more severe disc herniation was found in asymmetrical MCs. As common knowledge, nucleus pulposus had immunogenicity and caused autoimmune response which stimulated inflammation of the bone marrow. Nucleus pulposus induced the formation of many different inflammatory factors and then led to MCs. Conservative rehabilitation rate in patients with asymmetrical MCs was 47.06%, while this rate in patients with conventional MCs was 63.79%. Asymmetrical MCs indicated poor results in conservative treatment. More patients with asymmetrical MCs was diagnosed as cervical spondylotic radiculopathy and chose conservative treatment. That was why the final operation rate was similar between two kinds of MCs, even though the conservative rehabilitation rate was lower in patients with asymmetrical MCs.

Li et al. [25] observed 106 patients who underwent one-level ACDF from C4 to C7 and found although significant clinical improvement was showed in all three types of MCs, the outcomes of type 1 were better. In another study of Li et al. [26], he found that Modic-2 changes at adjacent level negatively impacted axial symptoms intensity and adjacent segment disease, but did not affect fusion rate and functional outcome In the current study, surgical treatment provides satisfactory results in both kinds of MCs and there was no difference in outcomes between two kinds of MCs after surgical. Anterior approach rate and posterior approach rate was similar in both groups. Adequate nerve decompression was the key to achieve good clinical prognosis. We did not compare the outcomes among three MCs types because of less patient number with MCs type 1 and type 3.

This study has some limitations. First, this study was limited by its retrospective nature. Second, pathogenesis and histological changes were not included in the current study. Finally, this was a single-center study and the number of patients was relatively small. In the future, the prospective, multicenter, and large-scale studies should be performed to confirm the results.

## Conclusions

C5/6 was the most commonly affected level by Modic changes. Disc herniation and nerve root compression symptom were more closely correlated with asymmetrical MCs than conventional MCs. Asymmetrical MCs indicated poor result in conservative treatment; however, the final operation rate was similar between the two kinds of MCs. The outcomes of surgical treatment were satisfactory both in patients with asymmetrical MCs and conventional MCs.

## Abbreviations

ACDF: Anterior cervical diskectomy and fusion
CIs: Confidence intervals
JOA: Japanese Orthopaedic Association
MCs: Modic changes
MRI: Magnetic resonance imaging
NDI: Neck Disability Index
OPLL: Posterior longitudinal ligament
ORs: Odds ratios
